# Blood eosinophil percentage as a predictor of response to inhaled corticosteroid in bronchiectasis

**DOI:** 10.1111/crj.13624

**Published:** 2023-04-26

**Authors:** Wang Chun Kwok, Terence Chi Chun Tam, David Chi Leung Lam, Mary Sau Man Ip, James Chung Man Ho

**Affiliations:** ^1^ Department of Medicine The University of Hong Kong Pokfulam China; ^2^ Department of Medicine Queen Mary Hospital Pokfulam China

**Keywords:** bronchiectasis, bronchiectasis exacerbation, eosinophil, inhaled corticosteroid, phenotype

## Abstract

**Introduction:**

The role of inhaled corticosteroid (ICS) among patients with bronchiectasis remains controversial. There is limited evidence of using baseline eosinophil count (absolute and percentage) as a marker to predict the role of ICS among patients with bronchiectasis.

**Methods:**

A retrospective case–control study was conducted in a major regional hospital and tertiary respiratory referral centre in Hong Kong, including 140 Chinese patients with noncystic fibrosis (CF) bronchiectasis, to investigate the exacerbation risks of bronchiectasis among ICS users and nonusers with different baseline eosinophil counts.

**Results:**

ICS user had significantly lower risk to develop bronchiectasis exacerbation with adjusted odds ratio (OR) of 0.461 (95% confidence interval [CI] 0.225–0.945, *p*‐value 0.035). Univariate logistic regression was performed for different cut‐offs of blood eosinophil count (by percentage) from 2% to 4% (with a 0.5% grid each time). Baseline eosinophil 3.5% was found to be the best cut‐off among all with adjusted OR of 0.138 (95% CI = 0.023–0.822, *p*‐value = 0.030).

**Conclusion:**

Baseline eosinophil count of 3.5% might serve as a marker to predict the benefits of ICS on exacerbation risk among patients with non‐CF bronchiectasis.

## Graphical abstract

Baseline blood eosinophil count of ≥3.5% might be a marker to predict the benefits of inhaled corticosteroid (ICS) on preventing exacerbation among patients with bronchiectasis.

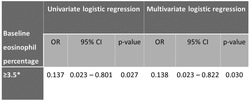

## 1 INTRODUCTION

Bronchiectasis is the end result of airway insults and predisposing conditions that culminate in airway injury, recurrent or persistent airway infections and destruction.^1^ It is also one of the commonest suppurative respiratory diseases. Noncystic fibrosis (CF) bronchiectasis is characterised by airway colonisation with micro‐organisms as well as infective exacerbations.

As a disease with hallmarks of chronic airway inflammation, inhaled corticosteroid (ICS) is used in patients with bronchiectasis for its local anti‐inflammatory effects and minimal systemic side effects. Despite the early reports of the potential benefits of ICS treatment in bronchiectasis,^2^, ^3^, ^4^, ^5^ Cochrane review concluded that there was insufficient evidence to support the routine use of ICS in adults with stable‐state bronchiectasis.^6^ The role of ICS in bronchiectasis is still controversial, especially when ICS treatment is not without risks.^7^, ^8^, ^9^

To provide personalised treatment, phenotyping of airway diseases is crucial,^10^, ^11^, ^12^, ^13^, ^14^, ^15^, ^16^ and blood eosinophil count is one of the most commonly used biomarkers in phenotyping.^14^, ^15^, ^16^ Eosinophilic phenotype is also reported in bronchiectasis,^17^, ^18^, ^19^, ^20^ and it may play a role in the therapeutic options in bronchiectasis,^21^, ^22^ especially on ICS treatment.^23^, ^24^ Blood eosinophil count may also predict the preferential localisation of bronchiectasis in patients with severe asthma, and type 2 inflammation was postulated to have causative role in the development of bronchiectasis among severe asthma patients.^25^, ^26^

Apart from the lack of quality evidence from planned large scale studies, this study aims to find out the exact predictive biomarker (absolute versus relative percentage blood eosinophil count), and the optimal cut‐off that might define clinical benefit from ICS in bronchiectasis remains controversial. We hypothesise that blood eosinophil level could predict the benefits from ICS in patients with bronchiectasis.

## 2 MATERIALS AND METHODS

This was a retrospective case–control study. Patients with bronchiectasis on ICS treatment in the designated bronchiectasis clinic at Queen Mary Hospital, Hong Kong, from year 2000 to 2021, were included. Patients with bronchiectasis but not on ICS in the study period were included as the control group. The controls were individually matched at 1:1 ratio by age (±5 years), gender, smoking status (never‐smokers vs. ever‐smokers), history of exacerbation in past 1 year (yes or no) and severity of bronchiectasis (*Pseudomonas aeruginosa* chronic infection and number of lobes involved). If more than one suitable control fulfilling the matching criteria is found, random selection would be performed by computer software.

Queen Mary Hospital is one of the major regional hospitals in Hong Kong and a University‐affiliated tertiary referral respiratory centre, with a designated bronchiectasis clinic for managing patients with non‐CF bronchiectasis of different disease severity. The investigators reviewed clinic records and radiographic findings to validate the diagnosis of bronchiectasis. Patients' records were accessed through the Electronic Patient Record (ePR) system of the Hong Kong Hospital Authority, which comprises both out‐patient and in‐patient episodes. The information available includes demographics, clinical notes, investigation results and treatments.

The inclusion criteria include Chinese patients with non‐CF bronchiectasis age at or above 18 years old. The diagnosis of bronchiectasis is confirmed; it meets the international consensus recommendations on the criteria and definitions for the radiological and clinical diagnosis of bronchiectasis in adults.^27^ The radiological criteria include an inner airway–artery diameter ratio of 1:5 or more, an outer airway–artery diameter ratio of 1:5 or more or a lack of tapering of the airways and visibility of airways in the periphery. The clinical criteria include at least two of the following symptoms: (1) a cough most days of the week, (2) sputum production most days of the week and (3) a history of exacerbations.^28^ Exclusion criteria included co‐existing asthma (compatible clinical history with wheeze, shortness of breath, chest tightness, cough that vary over time and intensity; previous physician diagnosis of asthma; with significant bronchodilator response on spirometry be a supporting criteria for cases in doubt), COPD (significant smoking history, compatible clinical history, spirometry with irreversible airflow obstruction, with or without radiological evidence of emphysema), traction bronchiectasis from interstitial lung disease and individual lost to follow‐up. Demographic data (age, gender, smoking status), clinical data/investigations (aetiology of bronchiectasis, comorbidities, treatment records, spirometry results, sputum culture results) and use of ICS (type and dose) were retrieved from clinical records. Regular use of ICS was defined as continuous use for at least 12 months within the study period. Baseline blood eosinophil level was the value taken at clinically stable state, which is defined as at least 90 days free from exacerbation, antibiotics and systemic steroid exposure. Among ICS group, the blood eosinophil level immediately before ICS commencement was taken as the baseline value. In the non‐ICS group, the blood eosinophil level taken at first clinic assessment at stable state was chosen to be the baseline value. Eosinophil percentage at stable state was also calculated.

The primary outcome was bronchiectasis exacerbation within 1 year of follow‐up period. The starting date of the assessment was the date of initiation of ICS for the ICS group. The time from first specialist consultation to ICS initiation was calculated for each case. The interval of each case was matched with the corresponding control to define the starting date of assessment of the control. Bronchiectasis exacerbation was defined as (1) a deterioration in three or more of the key symptoms (including cough, sputum volume and/or consistency, sputum purulence, dyspnea and/or exercise tolerance, fatigue and/or malaise and hemoptysis) for at least 48 h and (2) clinician's assessment that a change in treatment was required with systemic (oral or parenteral) antibiotics course being prescribed as deemed necessary after clinicians consultation.^23^ Patients who had bronchiectasis exacerbations that required in‐patient care during the follow‐up period were identified from the ePR. The study was approved by the Institutional Review Board of the University of Hong Kong and Hospital Authority Hong Kong West Cluster (UW 22‐578).

### 2.1 Statistical analysis

The demographic and clinical data were described in actual frequency or mean ± SD. Baseline demographic and clinical data were compared between the two groups (with or without ICS treatment) with independent *t*‐tests. Logistic regression was used to estimate the risk of bronchiectasis exacerbation and pneumonia among ICS and non‐ICS users in the 1‐year follow‐up period. FACED scores (Forced expiratory volume in 1 second (FEV1) in percentage predicted [F], Age [A], Chronic colonization by *Pseudomonas aeruginosa* [C], Extension of the disease by radiological assessment [E], Dyspnoea [D]) and exacerbation history in prior 1 year were adjusted as a potential confounder. To define the optimal cut‐off of eosinophil level, univariate logistic regression was performed for different cut‐offs of blood eosinophil count at a fixed grid increment. Statistical significance was determined at the level of *p* = 0.05. All statistical analyses were performed using the 26th version of SPSS statistical package.

## 3 RESULTS

A total of 140 Chinese patients with non‐CF bronchiectasis managed in Queen Mary Hospital (QMH) were included. Half of them received regular ICS.

### 3.1 Baseline characteristics

The mean age was 68.1 ± 11.2 years. There were more females (77%) and never‐smokers (90%). *P. aeruginosa* was the most common micro‐organism identified in sputum (51.4%), followed by 23 (16.5%) who had nontuberculous mycobacteria colonisation. The mean FEV_1_ was 1.53 ± 0.65 L (80 ± 24%). Multilobar involvement, defined as those with disease in more than three lobes, was seen in 84 (60%) of the patients. There were 48 patients who developed bronchiectasis exacerbation during the follow‐up period. The median time of ICS treatment is 8.64 years (interquartile range [IQR]: 4.44–14.2 years). The results are summarised in Table 1.

**TABLE 1:** Baseline demographic and clinical characteristics.

	Non‐ICS user (*n* = 70)	ICS user (*n* = 70)	*P*‐values
Age (years), mean ± SD	67.5 ± 11.0	68.6 ± 11.4	0.553
Male	16 (22.9%)	16 (22.9%)	1.0
Smoking status			1.0
Ever‐smoker	7 (10%)	7 (10%)	
Nonsmoker	63 (90%)	63 (90%)	
FEV_1_ (L)	1.66 ± 0.70	1.44 ± 0.60	0.114
FEV_1_ (% predicted)	86.0 ± 21.6	75.6 ± 25.1	0.032*
FVC (L)	2.32 ± 0.88	2.18 ± 0.74	0.418
FVC (% predicted)	93.6 ± 22.1	91.1 ± 19.4	0.261
FEV1/FVC ratio (%)	72.8 ± 7.4	66.7 ± 13.1	0.080
Bronchodilator reversibility (mL)	68.5 ± 39.8	58.2 ± 57.1	0.392
Bronchodilator reversibility (%)	4.1 ± 2.6	4.1 ± 4.3	0.995
Extent of involvement ≥3 lobes	41 (58.6%)	43 (61.4%)	0.730
*Pseudomonas aeruginosa* chronic infection	35 (50.0%)	37 (52.9%)	0.735
Nontuberculous mycobacteria colonisation	15 (21.4%)	8 (11.4%)	0.09
FACED score, median (IQR)	4 (3–5)	4 (3–5)	0.434
Exacerbation(s) in the past 1 year	55 (78.6%)	50 (74.1%)	0.329
Number of exacerbations in the past 1 year			0.057
No	15 (21.4%)	20 (28.6%)	
1	52 (74.3%)	30 (42.9%)	
2	2 (2.9%)	14 (20.0%)	
3	1 (1.4%)	6 (8.6%)	
Hospitalised exacerbation(s) in the past 1 year	23 (32.9%)	20 (28.6%)	0.148
0	47 (67.1%)	50 (71.4%)	
1	23 (32.9%)	16 (22.9%)	
2	0 (0%)	3 (4.3%)	
3	0 (0%)	1 (1.4%)	
Number of exacerbations in the follow‐up period of 1 year			0.054
0	40 (57.1%)	52 (74.3%)	
1	22 (31.4%)	8 (11.4%)	
2	3 (4.3%)	6 (8.6%)	
3	3 (4.3%)	2 (2.9%)	
4	2 (2.9%)	2 (2.9%)	
Number of hospitalised exacerbations in the follow‐up period of 1 year			0.357
0	62 (88.6%)	64 (91.4%)	
1	6 (8.6%)	2 (2.9%)	
2	2 (2.9%)	3 (4.3%)	
3	0 (0%)	1 (1.4%)	
Pneumonia in the follow‐up period of 1 year	4 (5.7%)	6 (8.6%)	0.512
*Pseudomonas aeruginosa* detection upon follow‐up (%)	29 (41.4%)	37 (52.9%)	0.176
Baseline blood eosinophil count (× cells/μL) (median, IQR)	120, 105	120, 140	0.386
Baseline blood eosinophil % (median, IQR)	2.06, 2.28	2.08, 2.24	0.707
Hypertension	18 (26.1%)	25 (35.7%)	0.220
DM	7 (10.1%)	8 (11.4%)	0.807
Hyperlipidemia	6 (8.7%)	6 (8.7%)	0.841
IHD	6 (8.7%)	6 (8.7%)	0.979
Stroke	5 (7.2%)	2 (2.9%)	0.237
AF	7 (10.1%)	6 (8.6%)	0.750
ICS use and daily dose			
Fluticasone propionate	‐	39 (55.7%)	
200 μg		20 (51.2)	
500 μg		10 (25.6%)	
1000 μg		9 (23.1%)	
Budesonide	‐	18 (25.7%)	
320 μg		4(22.2%)	
400 μg		5 (27.8%)	
640 μg		8 (44.4%)	
1280 μg		1 (5.6%)	
Beclomethasone	‐	13 (18.6%)	
400 μg		3 (23.1%)	
800 μg		10 (76.9%)	
Prophylactic regular intravenous antibiotics	2 (2.9%)	6 (8.6%)	0.145

Abbreviations: AF, atrial fibrillation; DM, diabetes mellitus; FEV_1_, forced expiratory volume in one second; FVC, forced vital capacity; ICS, inhaled corticosteroid; IHD, ischemic heart disease; IQR, interquartile range; mL, millilitre; SD, standard deviation.

* Statistically significant.

### 3.2 Risk of bronchiectasis exacerbation among ICS users and non‐ICS users

Univariate regression analysis showed that the ICS users had significantly lower risk to develop bronchiectasis exacerbation with odds ratio (OR) of 0.462 (95% confidence interval [CI] 0.226–0.944, *p* = 0.034). The result remained significant after adjusting for FACED score and exacerbation history in prior 1 year, with OR of 0.459 (95% CI 0.223–0.943, *p* = 0.034).

### 3.3 Risk of pneumonia among ICS users and non‐ICS users

Univariate regression analysis showed that the ICS users had insignificantly increased risk to develop pneumonia with OR of 1.547 (95% CI 0.417–5.739, *p* = 0.514).

### 3.4 Risk of bronchiectasis exacerbation among patients with different baseline blood eosinophil level by percentage

Further analysis was conducted to assess the potential benefits of ICS on exacerbation risk among patients with different baseline eosinophil levels by percentage which might be relevant to clinical benefit from ICS. Univariate logistic regression was performed for different cut‐offs of blood eosinophil count from 2% to 4% (with a 0.5% grid increment). Baseline blood eosinophil ≥3.5% was found to be the best cut‐off among all with OR of 0.137 (95% CI = 0.023–0.801, *p* = 0.027) that favoured ICS use with lower exacerbation risk. In multivariate logistic regression adjusted for FACED score and exacerbation history in prior 1 year, the risk of hospitalised bronchiectasis exacerbation remained significantly different, with OR of 0.132 (95% CI = 0.021–0.814, *p* = 0.029). The results are illustrated in Table 2.

**TABLE 2:** Risks of bronchiectasis exacerbation among ICS users and non‐ICS users at different cut‐offs of blood eosinophil percentage at stable state.

Baseline eosinophil percentage	No. of subjects (percentage on ICS)	Univariate logistic regression			Multivariate logistic regression^a^		
		OR	95% CI	*p*‐value	OR	95% CI	*p*‐value
≥2.0	74 (50%)	0.471	0.174–1.278	0.139			
≥2.5	52 (44%)	0.342	0.100–1.172	0.088			
≥3.0	46 (43%)	0.292	0.076–1.114	0.071			
≥3.5^b^	32 (47%)	0.137	0.023–0.801	0.027	0.132	0.0293–0.814	0.029
≥4.0	24 (54%)	0.218	0.032–1.485	0.120			

Abbreviation: ICS, inhaled corticosteroid.

^a^ Adjusted for FACED score and exacerbation history in prior 1 year.

^b^ Statistically significant after adjusted for FACED score.

### 3.5 Risk of bronchiectasis exacerbation among patients with different baseline blood eosinophil level by absolute count

Analysis was conducted to assess the potential benefits of ICS on exacerbation risk among patients with different baseline eosinophil levels by absolute count. Univariate logistic regression was performed for different cut‐offs of blood eosinophil count from 100 to 300 cells/μL (with a 50 cells/μL grid increment). Univariate logistic regression did not identify a cut‐off that predicts reduced risks of bronchiectasis exacerbation risk with ICS use. The results are illustrated in Table S1.

## 4 DISCUSSION

In this single centre study, baseline eosinophil count ≥3.5% was found to be a possible marker to enrich the benefit of ICS in reducing bronchiectasis exacerbation. Our finding, together with others in the literature, suggested that appropriate phenotyping of bronchiectasis with baseline blood eosinophil count by percentage may maximise the clinical benefit of ICS use among patients with bronchiectasis.

In recent years, phenotyping of airway diseases based on the type of airway inflammation has emerged with insights towards personalised treatment.^10^, ^11^, ^12^, ^13^, ^14^, ^15^, ^16^ Peripheral eosinophilia is one well‐reported phenotype among airway diseases. In patients with chronic obstructive pulmonary disease (COPD), baseline blood eosinophil count has become a reliable biomarker to guide the initiation of ICS, despite the lack of clinical benefit for all‐comers.^14^, ^15^, ^16^ A multicohort study in Europe reported an approximate 20% prevalence of peripheral blood eosinophilia in bronchiectasis. Adjusted for infection status, elevated blood eosinophil counts of 100–300 cells/μL and >300 cells/μL were associated with a shorter time to exacerbation of bronchiectasis, compared with those having eosinophil count <100 cells/μL.^17^ A Greek study noted that 17.5% of patients with bronchiectasis might have an eosinophilic phenotype (defined as the presence of more than 3% sputum eosinophil at stable state) which was associated with higher levels of fractional exhaled nitric oxide (FeNO), greater bronchodilator reversibility in forced expiratory volume in 1 second (FEV_1_) and higher sputum IL‐13 levels.^18^ Another Spanish study found that patients with blood eosinophil levels above 100 cells/μL had milder diseases with better clinical outcomes, lung function and nutritional status, while systemic inflammatory levels were lower compared with their noneosinophilic counterparts with bronchiectasis.^19^ The role of ICS among patients with different phenotypes of bronchiectasis based on the inflammatory pattern remains controversial, despite growing evidence in this area. In a pooled post hoc analysis of two randomised clinical trials, they tested on various cut‐off of eosinophil count as in the percentage and assessed if that can predict the benefits of ICS among patients with bronchiectasis. Martinez‐Garcia et al. identified that at the cut‐off of >3% and >4%, ICS usage can reduce exacerbations and hospitalisation in patients with bronchiectasis.^20^ Another unplanned post hoc analysis of a randomised, double‐blind, controlled study also suggested that 6‐month treatment with inhaled fluticasone propionate in adults with bronchiectasis significantly improved quality of life with a trend of lower exacerbation rate among those with a blood eosinophil count ≥3% or ≥150 cells/μL compared with control.^21^ Nonetheless, post hoc analysis of another randomised, double‐blind, parallel‐group trial of 40 patients with bronchiectasis revealed no significant change in quality of life after 6 months of inhaled budesonide treatment in the subgroup with baseline blood eosinophil count of at least 3% and 150 cells/μL.^22^

The role of ICS in preventing bronchiectasis exacerbation remained controversial. Despite the evidence from earlier reports, this was challenged by subsequent Cochrane review and European guidelines. Until recently, phenotyping in airway diseases has gained popularity and become the rationale to revisit the role of ICS in bronchiectasis of different phenotypes. While neutrophilic inflammation was thought to be the leading pathogenic mechanism in bronchiectasis, there has been growing evidence that a subgroup of patients had eosinophilic inflammation.^17^, ^19^ In a European multicohort study, eosinophilic bronchiectasis, as defined by blood eosinophil counts of ⩾300 cells/μL, was associated with shortened time to exacerbation.^17^ The same also applied to COPD, which led to a change in the practice in terms of when to initiate ICS. Although those with eosinophilic phenotype were reported to have milder disease, they were still prone to exacerbate if they had other indicators of severity, as suggested by FACED score and bronchiectasis severity index (BSI). Identifying those with eosinophilic phenotype might help to reduce exacerbation risk with ICS treatment. Previous studies using blood eosinophil count (absolute or percentage) as predictive markers for the benefits of ICS in preventing bronchiectasis exacerbation were controversial. Various cut‐offs for baseline eosinophil count and percentage were used in the literature. In a study by Martinez‐Garcia et al., the cut‐offs of eosinophil percentage of 3% and 4% were used.^20^ Our study revealed similar findings, and a cut‐off at 3.5% baseline blood eosinophil could optimally enrich the benefit of ICS even after adjusting for FACED score.

On the contrary, blood eosinophil count at stable state did not predict the potential benefits of ICS use in bronchiectasis. This could be explained by the fact that as a disease with predominant neutrophilic airway inflammation, blood eosinophil level as expressed in percentage may better reflect the phenotype than absolute count in bronchiectasis. Using blood eosinophil percentage has the advantage as it includes blood neutrophil count as the denominator, which would be more reflect the inflammatory status and phenotype in bronchiectasis. This could explain why blood eosinophil percentage but not absolute count can predict the potential benefits of ICS use in bronchiectasis.

In contrast to previous reports, our study is characterised by having a more ethnically homogeneous population (Chinese only) without co‐existing asthma or COPD. As some patients with asthma and COPD are known to have blood eosinophilia which may affect the clinical course, excluding these concomitant airway diseases as in our study would allow a dedicated assessment of the role of ICS among bronchiectasis patients with blood eosinophilia phenotype. Furthermore, adjusting for severity with FACED score in our study would take into consideration the potential confounders of exacerbation risk, which was lacking in prior studies. Hence, we believe that our findings can better reflect the true effect from ICS use based on blood eosinophil count. The potential benefit from ICS in bronchiectasis with high blood eosinophil is also consistent with the findings in asthma and COPD, in which eosinophilic (rather than neutrophilic) inflammation favours response to ICS. Nonetheless, the best blood eosinophil count (absolute and percentage) that predicts ICS response in bronchiectasis is yet to be defined. Even in COPD and asthma, different blood eosinophil count and percentage cut‐offs were employed in different settings, as in the indications of different biologics for treating asthma. Our study, on top of existing evidence, could provide insights for future dedicated clinical trials of ICS in selected population of bronchiectasis.

There are a few limitations in our study. First, this study involved only a single centre. However, being a tertiary medical centre, the respiratory unit received referrals from all other health care facilities across the territory. Patients diagnosed with bronchiectasis were managed in a designated bronchiectasis clinic in our centre. Second, lung function tests were done at different time‐points for patients in this cohort. Despite this, the results from our study are consistent with previous reports in the literature. As a retrospective study, the dose and type of ICS used were not standardised. There may be a remote possibility that the potency of ICS used may be different in reducing the risk of exacerbation. A prospective study using standardised fixed dose ICS can certainly provide a more robust assessment on the effect of ICS in bronchiectasis exacerbation risk.

## 5 CONCLUSION

Baseline blood eosinophil count of ≥3.5% might be a marker to predict the benefits of ICS on preventing exacerbation among patients with bronchiectasis.

## AUTHOR CONTRIBUTIONS

Dr. Wang Chun Kwok was involved with study concept and design, analysis and interpretation of data, acquisition of data, drafting of manuscript and approval of the final version of the manuscript. Dr. David Chi Leung Lam, Terence Chi Chun Tam and Prof. Mary Sau Man Ip were involved with critical revision of the manuscript for important intellectual content and approval of the final version of the manuscript. Dr. James Chung Man Ho was involved with the study concept and design, drafting of manuscript, critical revision of the manuscript for important intellectual content, study supervision and approval of the final version of the manuscript.

## CONFLICT OF INTEREST STATEMENT

The authors have no conflicts of interest to disclose.

## ETHICS STATEMENT

The study was approved by the Institutional Review Board of the University of Hong Kong and Hospital Authority Hong Kong West Cluster (UW 22‐578). The requirement for informed consent is waived by the IRB.

## Supporting information


**Table S1.** Risks of bronchiectasis exacerbation among ICS users and non‐ICS users at different cut‐offs of blood eosinophil count at stable state

**Data S1.** Supporting Information

## Data Availability

Not applicable.
