# Supplementary material for: Blood eosinophil percentage as a predictor of response to inhaled corticosteroid in bronchiectasis
Source: Clin Respir J. 2023 Apr 26;17(6):548–55. doi: 10.1111/crj.13624 (PMC10265148; doi:10.1111/crj.13624)
Supplement: Supplementary file 1 — Table S1. Risks of bronchiectasis exacerbation among ICS users and non‐ICS users at different cut‐offs of blood eosinophil count at stable state [file CRJ-17-548-s001.docx]

**Supplementary table**

**Table S1.** Risks of bronchiectasis exacerbation among ICS users and non-ICS users at different cut-offs of blood eosinophil count at stable state

| **Baseline eosinophil count (cells/μL)** | **Univariate logistic regression** | | |
| --- | --- | --- | --- |
|  | **OR** | **95% CI** | **p-value** |
| **≥100** | 0.451 | 0.178 – 1.144 | 0.094 |
| **≥150** | 0.321 | 0.101 - 1.023 | 0.055 |
| **≥200** | 0.300 | 0.070 – 1.288 | 0.105 |
| **≥250** | 0.420 | 0.075 – 2.361 | 0.325 |
| **≥300** | 0.218 | 0.039 – 2.874 | 0.318 |
